# Baseline Comorbidities in a Population-Based Cohort of Rheumatoid Arthritis Patients Receiving Biological Therapy: Data from the Australian Rheumatology Association Database

**DOI:** 10.1155/2009/861481

**Published:** 2009-09-01

**Authors:** Andrew M. Briggs, Lyn March, Marissa Lassere, Christopher Reid, Lyndall Henderson, Bridie Murphy, Rosemarie van den Haak, Adam Rischin, Margaret Staples, Rachelle Buchbinder

**Affiliations:** ^1^Monash Department of Clinical Epidemiology, Cabrini Hospital, Malvern, Victoria, Australia; ^2^Department of Epidemiology and Preventive Medicine, Monash University, Alfred Hospital, Prahran, Victoria, Australia; ^3^School of Physiotherapy and Curtin Health and Innovation Research Institute, Curtin University of Technology, Perth, WA, Australia; ^4^Institute of Bone and Joint Health, University of Sydney, NSW, Australia; ^5^Department of Rheumatology, Royal North Shore Hospital, St Leonards, NSW, Australia; ^6^Faculty of Medicine, University of New South Wales, NSW, Australia; ^7^Department of Rheumatology, St George Hospital, Kogarah, NSW, Australia; ^8^Centre for Clinical Research Excellence in Therapeutics, Monash University, Mebourne, Victoria, Australia

## Abstract

*Aims*. To describe the baseline characteristics of an Australian population-based cohort of rheumatoid arthritis (RA) patients commencing biological therapy. *Methods*. Descriptive analysis from the Australian Rheumatology Association Database (ARAD). *Results*. Up to October 2006, there were 681 RA patients taking biologics enrolled in ARAD. Baseline data were available for 624 (72% female, mean (SD) age 57.0 (12.5) years). Of these, 59.5% reported at least one comorbid condition, most commonly hypertension (35.7%) and osteoporosis (30.4%); 61 (9.8%) had a history of malignancy (35 nonmelanoma skin, 5 breast, 4 bowel, 5 cervix, 3 melanoma, 3 prostate and 1 each of lip, lung, myeloma, testis, uterus, vagina). Self-reported infections within the previous 6 months were common (71.5%). *Conclusions*. History of comorbidities, including recent infections, is common among Australian RA patients commencing biologics, and 10% have a history of malignancy. This may impact future evaluations of health outcomes among this population, including attribution of adverse events of biologic therapy.

## 1. Introduction

Rheumatoid arthritis (RA) is a chronic, autoimmune, inflammatory joint disease, which if left untreated often results in progressive and irreversible joint damage and disability [1]. As well as being associated with premature mortality, it has a substantial impact upon quality of life, physical, social and emotional function [2]. In Australia, 2.4% of the population report having RA [3]. 

Biological drugs (bDMARDs) including etanercept, infliximab, adalimumab, anakinra, rituximab and abatacept have been introduced into clinical practice in Australia and elsewhere with the anticipation that they represent a significant breakthrough in terms of altering the course and prognosis of RA [4]. Preliminary, uncontrolled Australian data suggest that after three months of therapy, etanercept is well tolerated and of benefit for patients with severe active RA, although its long-term safety and effectiveness remains uncertain [5]. This uncertainty highlights the need for careful longitudinal observation in routine clinical care, ideally using population-based health registries. 

Health registries that aim to determine the long-term safety of new drug therapy including the incidence and risk factors for adverse events need to consider potential effect modifiers. Comorbidities are one example of an important effect modifier at baseline and over time. The impact of RA is complicated by the relatively high prevalence of comorbidities such as gastrointestinal disease, malignancy, infections, osteoporosis and cardiovascular disease [6, 7] from both iatrogenic and disease-related causes. Notably, comorbidity has been shown to be a major predictive factor for health outcomes in patients with RA [2, 8] with premature mortality largely attributed to cardiovascular disease, infection and malignancy [9, 10]. 

The importance of considering comorbidity is particularly relevant to Australian patients. Australian patients may have a greater prevalence of comorbidities given the strict disease status, severity and treatment response requirements, including multiple previous DMARD use, which need to be satisfied before bDMARDs can be prescribed under government-subsidised schemes (Table 1). Furthermore, like other jurisdictions, previous and/or current malignancy is not an absolute contraindication to prescribing bDMARDs in Australia [11], although the risk of certain malignancies may be dissimilar due to differences in population and environmental factors. For example, Australia has the highest incidence of skin cancer in the world [12]. 

The primary aim of this paper is to outline the baseline characteristics including history of malignancy and infection in a population-based cohort of patients commencing bDMARD therapy for severe, active RA using data from the Australian Rheumatology Association Database (ARAD) [13]. ARAD is a national observational database, established by the Australian Rheumatology Association (ARA) in 2003 preceded by a pilot study that commenced in 2001, to determine the long-term effectiveness and safety of bDMARDs for patients with inflammatory arthritis in routine clinical practice.

## 2. Methods

### 2.1. ARAD Design

Details about the structure, governance and content of ARAD have been described previously [13]. Briefly, ARAD is a voluntary registry that collects longitudinal health outcomes data from Australian patients with inflammatory arthritis (RA, psoriatic arthritis, ankylosing spondylitis and juvenile idiopathic arthritis) treated with biological drugs as well as a control group of similar patients who are not receiving biological therapy. Patients are either referred to ARAD by their treating rheumatologist or they can self-refer. Patients taking bDMARDs are most commonly referred to ARAD by their treating rheumatologist at the time of initiation of biological therapy but can enrol at any time and controls (those not prescribed biologics) can enrol at any time. To 11th December 2008, 3025 participants have been enrolled in ARAD. They include 2366 with RA, 389 with AS, 186 with psoriatic arthritis and 83 with juvenile idiopathic arthritis. Two hundred and one (75.2%) rheumatologists from all Australian states and territories have contributed patients. 

Ethical approval for ARAD has been granted by twenty committees and organisations across Australia (see the appendix). All participants provide written permission to be contacted by ARAD investigators and written informed consent to participate in the registry.

### 2.2. Data Collection

Data collected from the rheumatologist at the time of RA patient enrolment include diagnosis and disease status data (ESR, CRP and joint count (see Table 1 for PBS definition of joint count)) and the bDMARD prescribed (if applicable). All ARAD participants complete a detailed entry questionnaire and six-monthly follow-up questionnaires returned in a reply paid envelope. Returned data are scanned into the database via teleform and subject to rigorous quality control and data validation processes to ensure database quality. Data collected from the participants include: demographic details, disease duration and severity, self-reported past and current medical history including cancers and other chronic conditions, use of antirheumatic drugs, smoking and alcohol history, generic measures of quality of life including the Short Form-36 (SF-36) (subscale scores range 0–100, 100 = perfect health) [14], Assessment of Quality of Life (AQoL) (score range 0-1, 1 = full health) [15] and European Union Quality of Life (EuroQoL) ([16] and arthritis-specific disability assessed by the Health Assessment Questionnaire (score range 0–3, 0 = no disability)) [17]. 

For the purpose of this study, ARAD participants were included if they had RA according to their rheumatologist, had enrolled in ARAD prior to October 1, 2006, and had completed a baseline questionnaire prior to commencement of bDMARD therapy. Baseline characteristics, medical history and health-related quality of life of RA patients commencing bDMARDS were extracted from the last questionnaire completed prior to commencing bDMARD therapy. Some questions relating to baseline descriptive variables, comorbidity, and infection were only added to the baseline questionnaire in January 2006 and so were unavailable for participants who completed their baseline questionnaire prior to this time. For this reason, some of the data summarised in the results is based on a smaller sample size. Participants with a history of infection within the previous 6 months were asked to grade them as mild, moderate or severe, according to OMERACT guidelines [18]. An operational definition of the grading system is provided in the questionnaire (see footnote Table 6 for definitions). Current infections were defined as any infection experienced within the 6 months prior to completing the questionnaire. 

### 2.3. Verification of Malignancy

To verify all self-reported malignancies, the demographic details of all ARAD participants were matched to the National Cancer Statistics Clearing House (NCSCH) and the Victorian State Cancer Registry (VCR) in 2007. The NCSCH and VCR record details of all malignancies occurring in Australia (except Victoria) and Victoria respectively, apart from nonmelanoma skin cancers. Notification of malignancy to the state registries is mandatory by law and virtual complete ascertainment is achieved by notification from pathology laboratories, hospital medical record departments and by screening of death certificates. The International Classification of Diseases, 9th Revision (ICD-9), is used to code site of malignancy [19] and the ICD-O morphology rubrics to code histological type [20, 21]. Cancer registration became mandatory nationally (NCSCH) and in Victoria (VCR) in 1982. At the time of the study the NCSCH and VCR were complete between 1982 to 2003 and 1982 to 2005, respectively. 

Cancers prior to 1982 and after 2003/2005 for the NBSCH and VCR, respectively, were verified by histology report or confirmation by the treating doctor. The date of diagnosis of malignancy as recorded in the NCSCH and VCR was used in the analysis if available. Otherwise the date of the histology report or doctor verification was used.

### 2.4. Representativeness of ARAD Participants

To examine the extent to which ARAD includes a representative sample of Australian RA patients taking bDMARDs, we surveyed a random sample of 27 rheumatologists participating in ARAD (20% of those participating in mid 2006). The rheumatologists were asked to indicate whether all of their patients receiving a bDMARD had been enrolled in ARAD and reasons for nonenrolment.

### 2.5. Data Analysis

Responses to the health related quality of life instruments (SF-36, AQoL, EuroQoL, and HAQ) were coded according to the standard published algorithms as described by the developers [14–17]. Descriptive statistics were used for baseline characteristics and logistic regression was used to determine whether rheumatologists with a higher number of patients enrolled in ARAD were more likely to have enrolled all of their bDMARD patients. All data were analysed using SPSS for Windows, version 15.0 (Chicago, IL, USA).

## 3. Results

There were 681 RA patients who had taken bDMARDS and enrolled in ARAD between 1st September 2001 and 1st October 2006. bDMARDs prescribed were etanercept (*n* = 382, 56.1%), adalimumab (*n* = 253, 37.2%), infliximab (*n* = 39, 5.7%) and anakinra (*n* = 7, 1.0%). Fifty-seven (8.4%) RA patients had enrolled in ARAD after commencement of bDMARDs and were therefore not able to be included in this analysis. 

The baseline demographic and disease characteristics and health-related quality of life data for the remaining 624 (91.6%) patients are outlined in Table 2. Mean (SD) age of the cohort was 57.0 (12.5) years and 449 (72.0%) were female. At the time of commencement of bDMARDs, 572 (91.7 %) patients were receiving at least one DMARD (Table 2), most commonly methotrexate (60.1%), and 420 (67.3%) participants were taking prednisolone (or prednisone). Disease-specific and generic health-related quality of life was significantly impaired (Table 2).

At baseline, 56.8% of the cohort reported at least one comorbid condition with 23.9% reporting two and 6.1% reporting three or more. The most frequently self-reported past or current comorbidities (occurring in a fifth or more of the cohort) were hypertension (40.7%), osteoporosis (31.0%), hypercholesterolemia (26%), gastrointestinal disease (20.4%), eye disease (20.3%) and depression (19.6%) (Table 3). 

Sixty-five out of 624 participants (10.4%) had a verified history of malignancy prior to commencement of bDMARDs. These included nonmelanoma skin (*n* = 78 in 39 participants), breast (*n* = 6), cervix (*n* = 5), bowel (*n* = 3), prostate (*n* = 3), melanoma (*n* = 3), lip (*n* = 1), lung (*n* = 1), myeloma (*n* = 1), uterus (*n* = 1), testis (*n* = 1) and vagina (*n* = 1). The median time between cancer diagnosis and starting biologics was 7.8 years (range 21 days to 33.5 years). 

One hundred and thirteen (71.5%) participants reported an infection in the 6 months prior to commencing bDMARDs (Table 4). Kidney/bladder/urine and bone/joint/muscle were the most commonly affected sites for severe infections and skin/nail and eye/ear/nose/throat the most commonly reported sites for mild and moderate infections. 

Thirteen of the 27 rheumatologists (48%) responded to the representativeness survey. They had collectively enrolled 138 patients to ARAD. Five (38.5%) had enrolled all their patients receiving bDMARDs to ARAD (*n* = 78 (56.5%)). The remaining 8 rheumatologists identified 47 patients receiving bDMARDs whom they had not enrolled. Of these, 12 (25.5%) were deemed unsuitable to participate either due to sickness (*n* = 1) or inadequate English/literacy skills (*n* = 11); 19 (40.4%) had been invited to participate but declined; 16 (34.0%) were not enrolled due to other reasons (rheumatologist time constraints or hospital initiated therapy (*n* = 9), ARAD not discussed with the patient (*n* = 1), patient overseas (*n* = 1); patient undecided at the time of the survey (*n* = 5). Having more patients enrolled in ARAD was not associated with a higher odds of having enrolled all patients in ARAD (Odds Ratio 1.10 (95% CI 0.96 to1.25)).

## 4. Discussion

Our study has found that more than half of the RA patients who commence bDMARD therapy in routine care report having at least one comorbid condition while almost a quarter report having two or more. While it is not possible to directly compare comorbidity results between studies due to variability in defining comorbidity, the conditions included and the mode of data collection in the studies, our results are broadly consistent with previous reports of baseline status in other cohorts commencing biological therapy [2, 22, 23]. For example, the British Society for Rheumatology Biologics Registry (BSRBR) reported that 58% of the RA cohort had least one comorbid condition and 25% had more than one [22]. Similar results were reported in a Dutch cohort of RA patients taking bDMARDs (56% and 28%, resp.) [2]. On the other hand a lower proportion of patients (10.2%) were found to have a baseline concurrent medical condition in a Swedish cohort commencing bDMARD therapy [24] although these data were derived solely from the Swedish National Hospital Discharge Register and so are likely to be an underestimate. 

We were unable to compare our baseline comorbidity results with the characteristics of RA patients who have participated in randomised controlled trials as these trials do not report baseline comorbidities as a matter of routine and in any case comorbidities are often an exclusion criterion. However Zink et al. found that only 21–33% of RA patients from the German biologics register Rheumatoid Arthritis Observation of Biologic Therapy (RABBIT (in German)) would have been eligible for the major biologic trials that led to approval of the drugs [25]. Similarly, Sokka and Pincus estimated that only 5% of patients typically seen in practice would be eligible for inclusion into a bDMARD RCT [26].

The high prevalence of multiple medical conditions in RA patients commencing bDMARDs is likely to be multifactorial, including the effects of the disease and medications used to treat the disease. The most commonly reported medical conditions in RA patients commencing bDMARDs in our sample were hypertension, osteoporosis and depression. This is consistent with the BSRBR which found that hypertension and depression were the most common comorbidities present at baseline in RA patients commencing bDMARDs [22]. Hypertension has also been reported to be commonly present in cohorts of North American and Dutch patients commencing bDMARDs [2, 27] and depression is known to be a common consequence of rheumatoid arthritis [28, 29].

The overall prevalence of verified malignancy in our population-based cohort prior to commencement of bDMARDs (10.4%) is higher (1.5–3.0%) [2, 22, 27] or comparable (11.1%) [30], to previous population-based studies of RA but higher than the 3% reported in the BSRBR for people with RA commencing bDMARDs [2, 22, 27]. The most common malignancy was nonmelanoma skin cancers (*n* = 35) and there were also three verified melanomas. While we cannot determine whether this is higher than would be expected in the general population, an increased risk of skin cancer in individuals with RA relative to the general population has been reported previously [31]. The relative risk of skin cancer in patients with RA compared with the Swedish population was reported to be 1.66 (95% CI 1.50–1.84) [31]. Notably, after treatment with TNF inhibitors, the risk increased to 3.6 (95% CI 1.8–6.5). 

The prevalence of skin cancers in our cohort may also reflect the fact that Australia has the highest incidence of both melanoma and nonmelanoma skin cancers in the world [12]. For example, in 2002 nearly 2% of the whole Australian population, 4% aged ≥40 years and 8% aged ≥70 years, were treated for nonmelanoma skin cancer [32]. There were a substantial number of self-reported malignancies we were unable to verify for reasons such as they had occurred too long ago and/or the records were no longer available. These included 56 nonmelanoma skin cancers (some of which may have been removed without histological verification), suggesting that the estimated prevalence of skin cancers (and possible other malignancies) may be an underestimate. All new self-reports of skin cancer are now being systematically verified by histology and/or doctor report. This will provide important data about nonmelanoma skin cancer risk in RA patients exposed to biologics not available by any other means as these are not routinely notified to the Australian state cancer registries. 

We have previously reported an estimated 50% excess risk of malignancy amongst RA patients exposed to methotrexate relative to the general population (SIR = 1.5, 95% CI 1.2–1.9) [33]. We followed 459 methotrexate-treated RA patients in community practice in Melbourne Australia for a total of 4273 person-years (an average of 9.3 years) and relative to the general population found an increased risk of melanoma (SIR = 3.0, 95% CI 1.2–6.2) as well as nonHodgkin's lymphoma (SIR = 5.1, 95% CI 2.2–10.0) and lung cancer (SIR = 2.9, 95% CI 1.6–4.8). Further serial record linkage of ARAD to the Australian National Cancer Statistics Clearing House is planned to study melanoma and other cancer incidence and reoccurrence rates in the ARAD bDMARD cohort compared with the Australian general population over time and compared with an ARAD non-bDMARD control group. 

Infections of any severity in the 6-month period prior to commencing bDMARDs were reported by the majority (71.5%) of our sample. It is important to note that our severity grades differ from those of regulatory clinical trials and were specifically developed for patient self-report [18]. Moderate-grade infections were reported most commonly, involving predominantly the ear, nose and throat or skin/nails, while severe infections more commonly involved the skin/nails and genitourinary and musculoskeletal systems. An increased risk of serious infection has been reported among cohorts of patients receiving bDMARDs [9, 23] and the sites of serious infection observed in our cohort are similar to those reported in RA patients receiving bDMARD therapy [23]. Our baseline infection data collected prior to commencement of bDMARDs will enable comparison to the rates and types of infection that occur following commencement of bDMARDs. 

This paper is the first comprehensive description of comorbidities, including a history of infection and malignancy, among Australian patients with RA commencing biological therapy. Although the report characterises the profile of comorbidities among this population, our conclusions are limited by the lack of comparison with people with RA who are not receiving bDMARDs. At the present time, there are too few ARAD participants with RA who have not received a bDMARD to allow meaningful comparison. A further potential limitation of our study is that we did not verify the validity of patient self-report of comorbidities. The format in which possible comorbidities and infections were presented to ARAD participants is identical to how they are presented in this paper. We cannot be certain that ARAD participants understood every comorbidity and infection category. Furthermore, infection categories were not mutually exclusive, so participants were able to mark more than one infection which may have led to an overestimation of infection rates. 

Despite these questionnaire limitations, previous studies have found that self-report of comorbidities is reliable [34, 35]. Moreover, our earlier pilot investigations also provide some evidence of the validity of self-report among ARAD participants [36]. A final limitation is that we cannot be certain whether ARAD participants are representative of Australian patients who commence bDMARDs. Incomplete case ascertainment is an important consideration for data validity and generalisability of health registries. Where possible, all cases for a condition should be included in the registry to minimise the threat of selection bias which may skew results. Based upon the small sample of participating rheumatologists that were surveyed as part of this study, it appears that the majority of patients deemed suitable for ARAD enrolment are being invited to participate. This was recently verified in another survey we performed to assess patient and rheumatologist satisfaction with ARAD [37]. Further efforts are being directed towards assisting participating rheumatologists with patient enrolment to maximise case ascertainment and enhance the validity of the registry dataset.

## 5. Conclusion

Comorbidity is common among Australian patients with RA taking bDMARDs. This is an important consideration when evaluating the outcomes of bDMARD therapy and assigning attribution of adverse events. More than 70% of Australian RA patients commencing bDMARD therapy reported a history of infection in the 6 months preceding commencement of biologic therapy and 10% of patients initiating bDMARD therapy in Australia have a verified history of malignancy. Longitudinal follow up of these patients will be important to study the effects of bDMARDs on risk of cancer recurrence.

## Figures and Tables

**Table 1:** Disease status and treatment response criteria needed to be met by Australian patients in order to receive government-subsided bDMARDs.

Severe and active disease status	Failure to achieve an adequate response to treatment
(i) ESR ≥ 25 mm/hr and/or CRP ≥ 15 mg/L, (ii) a total active joint count of at least 20 active (swollen and tender) joints, or (iii) at least 4 active joints from the following list of major joints: (a) elbow, wrist, knee, and/or ankle (assessed as swollen and tender) and/or (b) shoulder and/or hip (assessed as pain in passive movement and restriction of passive movement, where pain and limitation of movement are due to active disease and not irreversible damage such as joint destruction or bony overgrowth)	(i) currently taking methotrexate at a dose of ≥7.5 mg/week (infliximab and anakinra only), (ii) failed to achieve an adequate response to methotrexate at a dose of ≥20 mg/week, (iii) failed to achieve an adequate response to methotrexate (≥7.5 mg/week) with 2 other DMARDs at approved doses, (iv) failed to achieve an adequate response following a minimum of 3 months treatment with Leflunomide alone, or Leflunomide with methotrexate, or Cyclosporin alone

**Table 2:** Baseline demographic and disease characteristics of the ARAD RA cohort (*n* = 624)*.

	Mean (SD)
Age (years)	57.0 (12.5)
Duration of RA (symptoms), years	16.0 (11.0)
Duration of RA (since diagnosis), years	14.4 (10.2)
ESR (mm/hr)	35.8 (26.8)
CRP (mg/L)	33.7 (38.2)
PBS tender/swollen joint count^#^	23 (12)
Overall pain due to arthritis in last week (0–100)	44.0 (25.8)
Overall patient global impression of disease activity in the last week (0–100)	42.0 (25.3)
Mean (SD) HAQ score (0–3, 0 no = disability) (*n* = 617)	1.8 (0.72)
Mean (SD) AQoL score (0-1, 1 = full health) (*n* = 613)	0.42 (0.24)
SF-36 score (0–100, 100 = perfect health) (*n* = 583)	
Physical component	28.05 (9.88)
Mental component	43.66 (12.57)
EuroQoL (UK weights) (0-1, 1 = perfect health) (*n* = 592)	0.46 (0.32)

	*N* (%)

Female	449 (72)
Smoking history	
Current	101 (16.2)
Past	266 (42.6)
Never	257 (41.2)

Alcohol consumption	
Never	178 (28.9)
Sometimes	373 (60.7)
Every day	64 (10.4)

At least one concomitant DMARDs	572 (91.7)
Methotrexate (oral or IM)	375 (60.1)
Salazopyrin	73 (11.7)
Leflunomide	166 (26.6)
Hydroxychloroquine	115 (18.4)
Cyclosporin	16 (2.6)
Azathioprine	9 (1.4)
Gold	8 (1.3)
Penicillamine	4 (0.6)

Currently taking prednisolone or prednisone	420 (67.3)

*n slightly different for some variables.

^#^Pharmaceutical Benefits Scheme (PBS) definition (see Table 1).

**Table 3:** Frequency of self-reported current and past comorbidities among rheumatoid arthritis patients commencing bDMARD (*n* = 624)^∗#^.

	*N* (%)
High blood pressure	254 (40.7)
Osteoporosis*	49 (31.0)
High blood cholesterol or lipids*	41 (26.0)
Gastrointestinal disease	127 (20.4)
Eye disease*	32 (20.3)
Depression*	31 (19.6)
Neurological disease*	27 (17.1)
Anaemia or blood disease	78 (13.5)
Diabetes	64 (10.3)
Heart attack or angina*	16 (10.0)
Lung disease	55 (8.8)
Thyroid*	10 (6.3)
Other heart disease (eg valve problems)	38 (6.1)
Coronary artery bypass graft/angioplasty/stent*	8 (5.1)
Mental illness other than depression	18 (2.9)
Liver disease	17 (2.8)
Stroke/Transient Ischaemic Attack (TIA)*	4 (2.5)
Kidney disease	13 (2.1)
Drug or alcohol abuse	6 (0.9)
Tuberculosis*	1 (0.6)
Verified malignancy	65 (10.4)
Number of participants reporting an infection within 6 months prior to commencing biological therapy*	113 (71.5)

*n = 58 for conditions indicated with an asterisk.

^#^Listed in order of descending frequency.

**Table 4:** Self-reported infections and their severity* within the six months prior to commencing bDMARDs (*n* = 158).

	Mild	Moderate	Severe
Skin or nail	16 (10.1)	14 (8.9)	8 (5.1)
Eye, ear, nose, throat	9 (5.7)	23 (14.6)	4 (2.5)
Heart	1 (0.6)	1 (0.6)	0 (0)
Chest or lung	2 (1.3)	7 (4.4)	1 (0.6)
Stomach, gut, gall bladder, liver	2 (1.3)	2 (1.3)	3 (1.9)
Kidney, bladder, urine	3 (1.9)	12 (7.6)	7 (4.4)
Bone, joint, muscle	0 (0)	5 (3.2)	6 (3.8)
Artificial joint	0 (0)	0 (0)	1 (0.6)
Brain or spinal cord	1 (0.6)	0 (0)	0 (0)
Blood	0 (0)	2 (1.3)	2 (1.3)
Viral	5 (3.2)	9 (5.7)	2 (1.3)
Other	0 (0)	2 (1.3)	4 (2.5)
Total infections	39 (24.7)	77 (48.7)	38 (24.0)

*Definitions of infection grades:

Mild: activities did not change because of complaint, did not see a doctor or require prescription treatment for the complaint;

Moderate: activities changed occasionally because of the complaint, saw a doctor and/or needed prescription medications to relieve the complaint;

Severe: caused a major change in activities, saw a doctor, required prescription medication which only provided partial relief; if a drug treatment was responsible it may have been stopped.

## Appendix

### List of Australian Institutional Human Research Ethics Committees

Cabrini Hospital, Melbourne; Northern Sydney Health; South Eastern Sydney and Illawarra Area Health; Australian Government Department of Veterans Affairs; Monash University; Royal Children's Hospital, Melbourne; NSW Population and Health Services Research Ethics; Tasmanian Human Research Ethics Committee; Australian Institute of Health and Welfare; Cancer Institute of NSW; Department of Health (CHIC ) Western Australia; St Vincent's Hospital, Melbourne; The Children's Hospital, Westmead; Department of Health South Australia Human Research Ethics Committee; Queensland Cancer Registry; the Tasmanian Cancer Registry; the Victorian Cancer Registry; the ACT Cancer Registry; Northern Territory Cancer Registry; the New South Wales Central Cancer Registry; the Western Australian Cancer Registry; the National Cancer Statistics Clearing House.
